# Remote monitoring of cancer patients during the Covid-19 pandemic – an interview study of nurses’ and physicians’ experiences

**DOI:** 10.1186/s12912-022-00953-8

**Published:** 2022-06-28

**Authors:** Vigdis Abrahamsen Grøndahl, Ann Karin Helgesen, Elisabet Holm, Jannik Magnussen, Ann-Chatrin Leonardsen

**Affiliations:** 1 Faculty of Health, Welfare and Organisation, Østfold University College, PB 700, NO-1757 Halden, Norway; 2 Østfold Hospital Trust, Postboks 300, NO-1714 Grålum, Norway

**Keywords:** Cancer patients, Covid-19, Healthcare personell, Interview, Nurses, Physicians, Qualitative analysis, Remote monitoring

## Abstract

**Background:**

Due to the Covid-19 pandemic, remote monitoring of patients outside hospitals rapidly increased. Previous studies show that healthcare professionals’ competence in digitalization needs to be improved. Little is known about how Covid-19 has affected the use of remote monitoring of cancer patients. The purpose of the study was therefore to explore healthcare personnels’ experiences with remote monitoring of cancer patients during the Covid-19 pandemic.

**Methods:**

The study had an explorative and descriptive design using semi-structured individual interviews for data collection. Data was analyzed by content analysis.

**Results:**

A total of ten healthcare personnel working in the cancer department and out-patient cancer clinic in the hospital participated; four physicians and six registered nurses. Two categories and four subcategories were identified: 1) «Maintaining personalized healthcare services» comprising the subcategories a) «Adjusting services to patients’ health condition» and b) «Ensuring continuity»; and 2) «A supplement, but not a replacement» comprising the subcategories a) «Impact on interpersonal relations» and b) «The importance of clinical assessment».

**Conclusions:**

This study indicates that remote monitoring through telephone was preferred by both healthcare personnel and patients. The nurses and physicians experienced a more frequent contact with their patients, but emphasized the importance of physical meetings for building relationship, and for thorough clinical examination. Our findings indicate a need to facilitate a work environment where healthcare personnel can be fast learners in using digital tools to provide best possible healthcare quality. Moreover, it is imperative to develop a workplace suitable for the use of digital technology for remote monitoring, and to provide digital tools that is easy to use for both healthcare personnel and patients.

## Background

Globally, cancer is the leading cause of death, and among the four leading causes of death before the age of 70 years [1]. Cancer disparities reflect the interplay among factors such as social determinants of health, behavior, biology, and genetics all of which can have profound effects on cancer risk and outcomes [2]. In addition, cancer incidence and mortality are rapidly growing world-wide with an expected increase of 47 percent in cancer incidence from 2020 to 2040 [1, 3].

The beginning of the Covid-19 pandemic in March 2020 led to an extreme pressure on already pressured healthcare services worldwide [4, 5]. The need to releave pressure on hospitals had an immediate impact on cancer care in many countries, with the delivery of treatment and care shifting from inhospital consultations to remote cancer patient monitoring [6, 7]. Digital solutions for patient management and monitoring were rapidly implemented as a replacement of person-to-person meetings due to the need to ensure infection control [8]. Moreover, healthcare personnel had in some cases to weigh the risk that the patients could be infected against the need for treatment and care in the hospital [9].

Studies have shown that digital competence among healthcare personnel might be a challenge [10–13]. In addition, healthcare personnels’ technological knowledge is a crucial determinant of whether technology is adopted or not [12, 14]. Hence, healthcare personnel will need competence beyond understanding how digital technology works to instruct patients in their use of technology [13].

The available remote health monitoring systems, their technologies, capabilities and actions vary to a large degree, and include both with-contact methods (sensors attached to body) and contactless methods (image-based and radar-based methods) [15]. Remote patient monitoring solutions can be grouped into three main categories: monitoring technologies such as mobile phones, smartphones, and tablets; store and forward applications, including systems that transmit clincial data to be analysed at a later date; and interactive solutions where healthcare personnel and patients can exchange information and communicate in real time [16]. Videoconferencing is the remote monitoring most frequently used [17].

The objectives of remote patient monitoring vary depending on the patients’ clinical condition, and include monitoring of a chronic condition to detect early signs of detoriation and prompt treatment and advice, provision of treatment or rehabiliation, education and advice for self-management, specialist consultations, real-time assessment of clinical status, and screening [16]. Remote patient monitoring has on one side been claimed to increase efficiency by allowing healthcare personnel to remotely educate and communicate with patients [18]. On the other side, technology supported healthcare may contribute to an increased distance between persons [19].

A systematic review on healthcare professionals’ experiences of digitalization concluded that competence in digitalization required strong professional knowledge and skills, that competence in digitalization was influenced by attitudes based on prior experiences, and that psychosocial and organisational factors were significant predictors [20]. Few studies have explored healthcare personells’ perspectives on remote monitoring of cancer patients as a consequence of the Covid-19 pandemic.

## Methods

### Aim

The aim was to explore healthcare personnels’ experiences with remote monitoring of cancer patients during the Covid-19 pandemic.

### Study design

The study had an explorative and descriptive design using individual in-depth interviews for data collection [21]. The manuscript adheres to the Consolidated criteria for Reporting Qualitative research (COREQ) [22].

### Setting of the study

The study was conducted in a medium-sized hospital trust in south-eastern Norway that serves approximately 322 000 inhabitants. The hospital has been recognized as a showcase of best practice in the implementation of healthcare technology, and placed in Europe’s Electronic Medical Record Adoption Model (EMRAM) stage 6 of 7 Stages Club by Healthcare Information and Management Systems Society (HIMSS) Europe, which is an organization that assesses and scores the clinical information technology systems in European hospitals [23]. The technology explored in this study was applied in order to streamline the hospital systems to limit the need for patients to attend to the hospital. Moreover, the technology includes solutions for distant monitoring of patients, through either mobile cell phones, video conferences, or software solutions included in tablets.

### Participants

A consecutive sampling method was used. Healthcare personnel working in the cancer department and out-patient cancer clinic in the hospital were invited to participate. Inclusion criteria were that they (1) gave their informed consent, (2) understood and were able to express themselves in Norwegian, (3) were authorized healthcare personnel, and (4) had used remote monitoring of cancer patients during the period from March 2020 and to the time of interview. A research assistant gave the healthcare personnel who met the inclusion criteria both oral and written information about the study. Those who signed the informed consent, were contacted by the researchers to make an appointment for the interview.

### Data collection and procedure

A semi-structured interview guide was developed based on relevant literature on healthcare personnels’ experiences with technology [20], as well as iterative discussions between the authors and two physicians working at the cancer department, until consensus was reached. Questions included the participants’ experiences with remote monitoring of cancer patients during the Covid-19 pandemic, the implications of remote monitoring on the relations with the patient and the relatives, and factors that the participant perceived promoted or inhibited the treatment and care when remotely monitoring the patient. Probing questions were used to deepen the understanding of the participants’ perspectives («can you please elaborate on that», «can you please give an example», «can you please tell me more about that»). In addition, demographic questions concerning age, gender, education and work experiences were gathered. Three of the authors (VAG, AKH, ACL), all experienced researchers, and unfamiliar to the participants, conducted the interviews. The interviews took place between December 2020 and February 2021. The participants were given the choice of whether the interview should be conducted as a video conference on a digital plattform (Skype/Zoom) or by telephone. All participants chose to conduct the interview by telephone, except one who wanted to meet the researcher in her office. The interviews lasted from 22 to 46 min (mean 34 min) and were digitally recorded. The interviews were transcribed verbatim by an external transcriber, who had signed a non disclosure agreement. Before and after each interview, the researchers wrote down their initial impressions and thoughts as a method of reflexivity [24] that were included throughout the analysis process.

### Data analysis

Conventional content analysis was used following the steps proposed by Hsieh and Shannon [25]. In step one the first author (VAG) read and re-read the transcripts to get a sense of the whole, and to familiarize with the data. In step two, the transcripts were read word by word, and words that captured key thoughts or concepts were higlighted in the text, and formed the initial codes (VAG). In step three, three of the authors (VAG, AKH, ACL) assessed the transcripts and the reflexivity notes, and discussed the initital codes. Through this process, labels for codes emerged that comprised more than one code. This initial coding scheme was discussed among all of the authors. In step four, the codes were sorted into categories based on how they were related or differed. The process went back and forth, to ensure that the categories were based on meaningfull clusters of codes. This was discussed among the authors until consensus was reached. See Table 1 for an example of the analysis process.

**Table 1:** Example of the analysis process

Transcript	Initial codes	Subcategories	Categories
P5: It depends on the patient’s situation, if it is uncomplicated and nothing new, then it’s ok to use the telephone P9: We have patients that will not recover.. they might need to see us face to face to feel safe	Use telephone when patient’s situation is ok Patients’ health condition decides how to monitor	Adjusting services to patients’ health condition	Maintaining personalized healthcare services

## Results

A total of ten healthcare personnel were invited and agreed to take part in the study; four physicians and six registered nurses (RNs). See Table 2 for an overview of the participants’ characteristics. The analysis revealed two categories. The first category was «Maintaining personalized healthcare services» with the subcategories «Adjusting services to patients’ health condition», and «Ensuring continuity». The second category was «A supplement, but not a replacement» with the subcategories «Impact on interpersonal relation» and «The importance of clinical assessment».

**Table 2:** The characteristics of the participants (*N* = 10)

Gender (*n* =)	
Female	9
Male	1
Age (years)	
Range	31—58
Mean	49,2
Position (*n* =)	
Registered nurse with specialisation in cancer nursing	6
Physicians with specialisation in oncology/hematology	4
Experience from the current workplace (years)	
Range	3—24
Mean	12

### Maintaining personalized healthcare services

To the participants, the most important thing was to ensure patients the same services as before the pandemic. They emphasized the importance of adjusting their services to the patients’ health condition at the specific point of time, and to use the type of remote technology the patients were able to use due to the consequences of their diagnosis, such as fatigue. Moreover, the participants reported a need to ensure continuity even though the pandemic limited the options for physical consultations.

#### Adjusting services to the patients’ health condition

All of the participants had used a mobile phone with and without video as well as tablets in remote monitoring of patients, both prior to and during the Covid-19 pandemic. Nevertheless, the participants described that they had to be fast learners of remote monitoring when the pandemic broke out, and the frequency of remote monitoring increased. This was both due to the fact that home visits were not allowed, and also, that the patients were afraid of being infected if they came to the hospital for their medical appointments. Participant 2 described:

«That was when we got up to speed with establishing the remote monitoring. So that we did not expose them to more risk by getting out of their homes if they did not have to».

The participants acknowledged that cancer patients’ health condition varies from day to day. Consequently, participants reported that some patients could use tablets in the beginning of their illness, but had to use the telephone in cases of deterioration, because they did not have the strenght to continue to communicate by writing on the tablets. Participant 1 described:

«We had met him at the ward and considered that it might be good for him to have the digital monitoring at home. It went well a couple of times, but then he got a lot sicker, and he tried to keep using the tablet, but in his situation, using telephone is a better alternative».

Participant 6 also added that using tablets gave the healthcare personnel the possibility to give more comprehensive information to the patients, allowing patients to read it repeatedly. Hence, the patients could read messages and respond when their health condition was at the best during the day. The tablet structured the communication between the patient and the healthcare personnel, which was seen as an advantage by the participants. All of the participants stated that using tablets was more like a one way communication, since they did not respond to the patients questions and answers until the next day, and not during weekends. This was seen as a drawback.

The participants said that talking with the patients using the telephone, gave them the possibility to give promptly feedback on the patients’ questions. The drawback was that the patient might forget the answers, being affected of medications or feeling ill, because it was only said verbally. One participant said that she always made a note after talking with the patients and had it sent to the patient to help them remember what they talked about. The participants also said that hearing the patients voice gave them much information about their health condition, that was important for them to personlize the treatment or services. Participant 1 further stated:

«The other (written communication through a digital platform) makes room for more interpretations. That is, when I look at an answer, and I am not sure, I can write back to the patient, but it takes time to get an answer, so it is much easier to clarify misunderstandings on the telephone ….».

#### Ensuring continuity

The participants described the outbreak of Covid-19 as a challenge for the continuity of their relationship with the patients. From one day to the next, the patients were only allowed in the department based on extensive deterioration of the health condition. In addition, the participants experienced that patients were afraid of being infected by Covid-19 at the hospital, and were reluctant or refused to come. Despite none, or less, possibility for physical meetings, the participants reported that the pandemic had led to more frequent appoinments with patients by telephone. Instead of less continuity, using telephone, enabled them to maintain or even improve the continuity in patient contact. Participant 4 illustrated.

«Instead of doing longtime planning for reduction of medicins, we make telephonecalls along the way, because many patients experiences symptoms when reducing the medication. By making the call more frequently, we can catch the symptoms much earlier and avoid side effects».

Continuity was described by all of the participants as an important factor when aiming for personalized care. They reported that the cancer department operated with a primary contact system, that allowed the primary contact nurse or physician to get to know the individual patient and the health condition, the patient’s relatives and their home situation. When patients called the hospital, and another nurse than the primary contact answered the telephone they emphasized linking up with a nurse or a physician that knew the patient. Participant 9 explained:

«We have a kind of primary nursing, but we discuss with our doctors, and also another nurse colleague, because it’s ok to be two that can assess the situation, and yes, that know the patient».

### A supplement, but not a replacement

The pandemic had forced the healthcare personnel to re-think how services may be provided. Even though the participants reported of several benefits from using remote monitoring of patients, they also emphasized that this service to them implicated a supplement and not a replacement. They all reported that remote monitoring included other aspects than the physical consultations, both regarding the nurse/physician–patient/relative relationship, and regarding the clinical assessment.

#### Impact on interpersonal relations

The participants experienced that physical meetings made it easier to remember the patient and their health problems, and probably for the patients to remember the physicians and nurses. Without an initial physical meeting the participants missed having a face associated with the voice, and that made the relationship different. To know the patients and having developed a close relationship prior to the pandemic, were expressed as important. Participant 4 elaborated:

«Some times I feel I do not really know the patient if I haven’t seen his face or how he act. Feel I may miss something … But with the patients I have seen recently, it’s no problem to use the telephone».

On the telephone, the participants experienced that the patients tended to say that their health status was better than it actually was, because it was difficult to make them relax during the conversation. On the other hand, one of the physicians also experienced that when the patients knew her, it was easy to get them to talk about their situation. But all participants expressed that face to face meetings made it easier to have deeper conversations. Participant 6 described it like this:

«Because when you meet in person, suddenly there is room for other topics than those planned. While when you call, …. well you save time, but loose, well, the important smalltalk that is created in a room with two people».

The participants also experienced that the technical equipment could be an additional disturbance for building a relationship if it didn’t work or if it was difficult for the patient or the healthcare personell to use. Technological challenges like problems logging in to the programme or that the video froze, had made the participants prefer to choose telephone rather than video consultations. Participants also perceived that older patients had a tendency to prefer telephone compared with younger patients who where more used to digital technology. They also experienced that the patients chose telephone rather than video consultations to avoid the physicians looking into their home, and also told about patients that had expressed that they needed to buy new furniture, before using video consultations. All participants also highlighted the importance of privacy when using videos for patient consultations. Participant 8 described:

«Even if I use the telephone, we need to have a place where we are not disturbed. And when I use Skype and the patient can see me and my surroundings, I do not want people to come and go. The patients get worried, and wow, who is that? Can they also hear and see me? No, it’s not ok».

Prior to the pandemic, the participants experienced that the patients were accompanied by their relatives when attending the hospital for medical appointments. The relatives were mostly seen as resources that would help the patients remember the information from the physicians and nurses. During the pandemic no relatives were allowed in hospital. The physicians emphasized that using video for remote monitoring of patients in their home made it easier to also include the relatives, because then they could also participate and be a resource for the patient. But when telephone was used instead of video, the physicians experienced that they did not know who were present, and that made the conversation more challenging. As Participant 8 said:

«And more people are sitting there, listening to me, and I demand to know who is present, since I cannot see them. And I make notes of who is present. And it is ok for them, because the relatives can not accompany the patient to a consultation in the hospital because of the pandemic, but at home they can particpate».

In addition, some of the participants reported that the relatives would sometimes take over for the patients and do the registrations on the tablets, when the patients did not have capacity to do so. One participant expressed that this could be sufficient in some situations, but sometimes she was not sure whether it was the patients’ feedback or the relatives’ feedback that was reported. Participant 6 elaborated:

«Ethically, it might sometimes be difficult, when decisions are made on behalf of a patient that is consent competent, but you have to talk to his or her spouse».

#### The importance of clinical assessment

The participants were worried that they might miss vital information if they were not able to clinically assess the patients. In a physical meeting, they could observe the patient’s body language, something they described was difficult when using the telephone. Some of the participants stated that using video expanded the possibilities for assessing the patients’ health status, but even so, both physicians and nurses preferred that the first meeting was at the hospital. Participant 5 said:

«It is not the same, because in a meeting, you read the body language, and try to make them comfortable, and it is difficult to gain a complete overview of how they feel if I have not seen them, and only met them on the telephone».

After the first physical meeting, the participants experienced that remote monitoring, mostly by telephone, was sufficient as a follow up as long as the patients’ health condition was stable and not deteriorating. But if the symptoms were increasing, they were afraid of not being able to identify all the symptoms by telephone or video. Participant 10 elaborated:

«We use the telephone a lot, and are encouraged to use videoconsultations more often, but it is not the same … I don’t think it is quality nursing either».

If the patients were to receive new or changed medication, the physicians expressed a need to meet the patient to make the right decision based on physical examination, also to make sure that the information was received and understood. The physicians were clear that they would not use the telephone or the tablet if they had bad news or information to give the patients. Then they preferred to meet face to face. They expressed the importance of being there for the patients and being able to comfort them, something they felt were difficult using telephone or video. Participant 7 stated:

«If I know the patient and their general condition well, and feel that it is ok to use the telephone without video, I do that, but there is always the unknown factor. It might be positive or negative, but you might miss it if you’re not in the same room and can look at the patient. I need to see the patient and observe the physical status, level of activity, to choose chemotherapy».

## Discussion

Our findings show that both physicians and nurses emphasized the importance of maintaining personalized healthcare services, continuously adjusting services to the patients’ health condition and focusing on continuity of care during the Covid-19 pandemic. Moreover, they highlighted that remote monitoring was experienced as a supplement, but not a replacement of person-to-person consultations.

Independent of the patients’ health condition, both personnel and patients preferred using a telephone. This is in-line with previous research stating that patients’ preferred method for communication with healthcare personnel was telephone calls [26], even if video-conferencing is most frequently used [17]. Maguire et al. (2021) [27] found that both healthcare personnel and patients experienced that a mobile phone-based symptom monitoring system was a positive addition to clinical care. Another study showed that healthcare personell continuosly individualized technology and work processes to meet the patients challenges when using video-conferencing in rehabilitation [28].

The participants in our study had limited experience with different digital remote monitoring systems, and described the outbreak of the pandemic as a starting point for fast learning. Research conclude that this shift in practice brought about by the pandemic, must be accompanied by improved training and awareness, enhanced infrastructure and evidence-based support to both healthcare personnel and patients if they are to harness the positive and offset the potentital negative consequences of the impact of Covid-19 on cancer care [9].

The participants were ambivalent regarding whether they had managed to ensure continuity of care during the pandemic. On one hand, they described that they had even more patient contact during the pandemic because it was easy to just make a telephone call rather than waiting for the next consultation. On the other hand, they were concerned of the consequences of the pandemic on the continuity of care. This is in line with Jacobs et al. (2017) [19] who claimed that healthcare supported by technology can be experienced as supportive, but also increasing the distance between career and patient at the same time.

The participants highlighted the importance of knowing the patient prior to using remote monitoring, and expressed concerns about the possibility of missing vital information when the conversations took place by telephone or tablet. This concern is known, and has led to the development of a brief tele-oncology communication guide (Comskil TeleOnc) to promote best practices in remote cancer care [17]. The communication guide comprises five steps; 1) establish the clinical-patient relationship, 2) set the agenda, 3) respond empathically to emotions, 4) deliver the information, and 5) end the televisit. The healthcare personnel in our study also commented on the possibility for privacy during digital patient meetings. They did not feel comfortable using an office where colleagues could interupt. How to protect patients’ privacy is highlighted in the first step of the Comskil TeleOnc communication guide [17].

Our participants were concerned with the technology itself, due to experiencing that the technology not always worked. A previous study found that the most important barriers to introducing technology in nursing homes were unstable technology and lack of support [29]. This included a lack of collaboration between the technological support service and healthcare personnel in contributing to create common values. Organizational issues, like user involvement, benefits, processes, technological support and leadership were among factors found in a systematic review, to be important when implementing new technology in healthcare services [30].

The healthcare personnel in our study stressed the importance that digitalization must be a supplement, and not a replacement, and all participants highlighted the importance of clinical assessment. Konttila et al. (2018) [20] emphasized that remote monitoring of patients require strong professional knowledge and skills. Further on, the World Health Organisation’s global strategy stresses that digital health should benefit people in a way that is ethical, safe, secure, reliable, equitable and sustainable [31]. The European Union (EU) [32] states that digitalization can support the reform of health systems and their transition to new care models centred on people’s needs and enable a shift from hospital-centred systems to more community-based and integrated care structures.

## Limitations

The study has some limitations that must be considered. Firstly, the sample size is small, and limited to one geographical area solely, which limits the transferability of our findings. We can not claim having achieved data saturation. Nevertheless, the data are rich, and reveals important experiences on remote monitoring of cancer patients during a pandemic from healthcare personnel’s perspectives. Secondly, the interviews were conducted by three different interviewers: the first, second and last author. Still, all three are experienced researchers and interviewers, and the interview guide was developed in collaboration between all authors. Thirdly, all interviews except one were conducted by telephone. The interviewers might have missed some of the participants reactions, because of lack of physical apperance. However, it is important for creating a relaxing interview situation that the participants’ wishes are met [21].

## Conclusions

The Covid-19 pandemic led to a rapid increase in use of remote monitoring of cancer patients, and the healhtcare personnel had to be fast learners. Even if digitalization has been seen as a solution, our findings indicate that both healthcare personnel and patients prefer using the telephone. The nurses and physicians experienced a more frequent contact with their patients, but emphasized the importance of the physical meeting, both for building a relationship with the patient, and when in need of a thorough clinical examination.

### Implications for clinical practice

Recommendations for clinical practice include: facilitate a work environment where healthcare personnel can be fast learners in using digital tools to provide best possible healthcare quality; enable a workplace suitable for the use of digital technology for remote monitoring; and provide digital tools that is easy to use for both healthcare personnel and patients.

### Future research proposal

To investigate the different stakeholder’s perspectives on remote monitoring can illuminate areas of importance for quality improvement and also guide further implementation of digital solutions for remote monitoring. The sample should include both cancer patients, their relatives, and hospital management.

## Data Availability

The datasets generated and/or analysed during the current study are not publicly available due to being collected in Norwegian, but are available from the corresponding author on reasonable request.
